# The role of gut microbiota in osteoporosis: underlying mechanisms, clinical associations, and emerging biomaterials

**DOI:** 10.3389/fimmu.2026.1828297

**Published:** 2026-05-20

**Authors:** Peng Ha, Ziying Wang, Guanlin Huo, Xiaomin Sun, Keqiang Yu

**Affiliations:** 1 Department of General Practice, General Practice Center, The Seventh Affiliated Hospital, Southern Medical University, Foshan, China; 2 Universiti Utara Malaysia, UUM Sintok, Kedah Darul Aman, Malaysia, UUM Sintok Kedah Darul Aman, Malaysia; 3 Affiliated Hospital of Changchun University of Chinese Medicine, Changchun, Jilin, China; 4 School of Traditional Chinese Medicine, Southern Medical University, Guangzhou,Guangdong, China

**Keywords:** biomaterials, bone metabolism, gut microbiota, gut-bone axis, osteoporosis, SCFAs (short chain fatty acids)

## Abstract

Osteoporosis is a prevalent metabolic skeletal disorder characterized by reduced bone mass, deteriorated trabecular microarchitecture, and increased fragility fracture risk, imposing substantial global medical, social and economic burdens. Current first-line antiresorptive and anabolic therapeutics are severely constrained by long-term adverse reactions, insufficient patient adherence, and compromised bone microenvironment remodeling capacity, leaving a large unmet clinical demand for multitargeted and translational interventions. The gut–bone axis has been recognized as a core interorgan regulatory signaling network, in which gut microbiota orchestrates bone homeostasis through multiple cascaded mechanisms, including microbial metabolite production (short-chain fatty acids, tryptophan derivatives and bile acids), osteoimmune balance modulation (Th17/Treg axis and macrophage polarization), intestinal barrier maintenance, as well as the regulation of estrogen bioavailability, calcium-phosphorus absorption and vitamin D/VDR signaling. In parallel, advanced functional biomaterials, including modified bone cements, injectable hydrogels, intelligent nanocarriers and immune-regulatory scaffolds, have overcome the defects of conventional bone grafts and inert implant materials, exhibiting tunable mechanical properties, controllable degradation and precise bioactive cargo delivery for osteoporotic bone repair. Notably, the emerging integration of biomaterial engineering with gut–bone axis microbiology has established an innovative “material–microbiota–metabolism–bone” therapeutic paradigm. rationally designed gut-targeted biomaterial platforms, such as metabolite-releasing nanoparticles, probiotic-encapsulated microcarriers and ion-doped multifunctional hydrogels, enable simultaneous local bone defect reconstruction and systemic intestinal microecology homeostasis regulation, thereby alleviating gut dysbiosis-derived chronic inflammation and preventing progressive bone loss. This review systematically elaborates the core molecular and pathological mechanisms by which gut microbiota regulates osteoporosis progression, summarizes the research advances and inherent limitations of traditional bone repair biomaterials, and highlights the latest progress of multifunctional biomaterials targeting gut–bone axis crosstalk. We further conduct a critical comparison of three mainstream administration routes (oral delivery, local bone delivery and systemic delivery) in terms of targeting efficiency, biosafety and clinical applicability, and clarify the translational trade-offs of different material-based strategies. Despite encouraging preclinical outcomes, the clinical translation of gut microbiota-modulating biomaterials remains hindered by individual microbial heterogeneity, long-term biocompatibility risks, and incomplete clarification of material–gut–bone interactive mechanisms. Collectively, this comprehensive review constructs a refined interdisciplinary framework and provides actionable theoretical guidance for the development of next-generation personalized, multi-pathway combined biomaterial therapies for osteoporosis.

## 1 Introduction

Osteoporosis is a metabolic bone disease defined by decreased bone mass, deterioration of bone microarchitecture, and increased skeletal fragility. These characteristics contribute to its high prevalence and significant associated disability (1).The clinical progression of osteoporosis is typically insidious and challenging to monitor over time (2, 3). Consequently, fragility fractures often occur, leading to severe consequences and imposing substantial medical, social, and economic burdens on affected individuals (2, 3).

**Figure 1:**
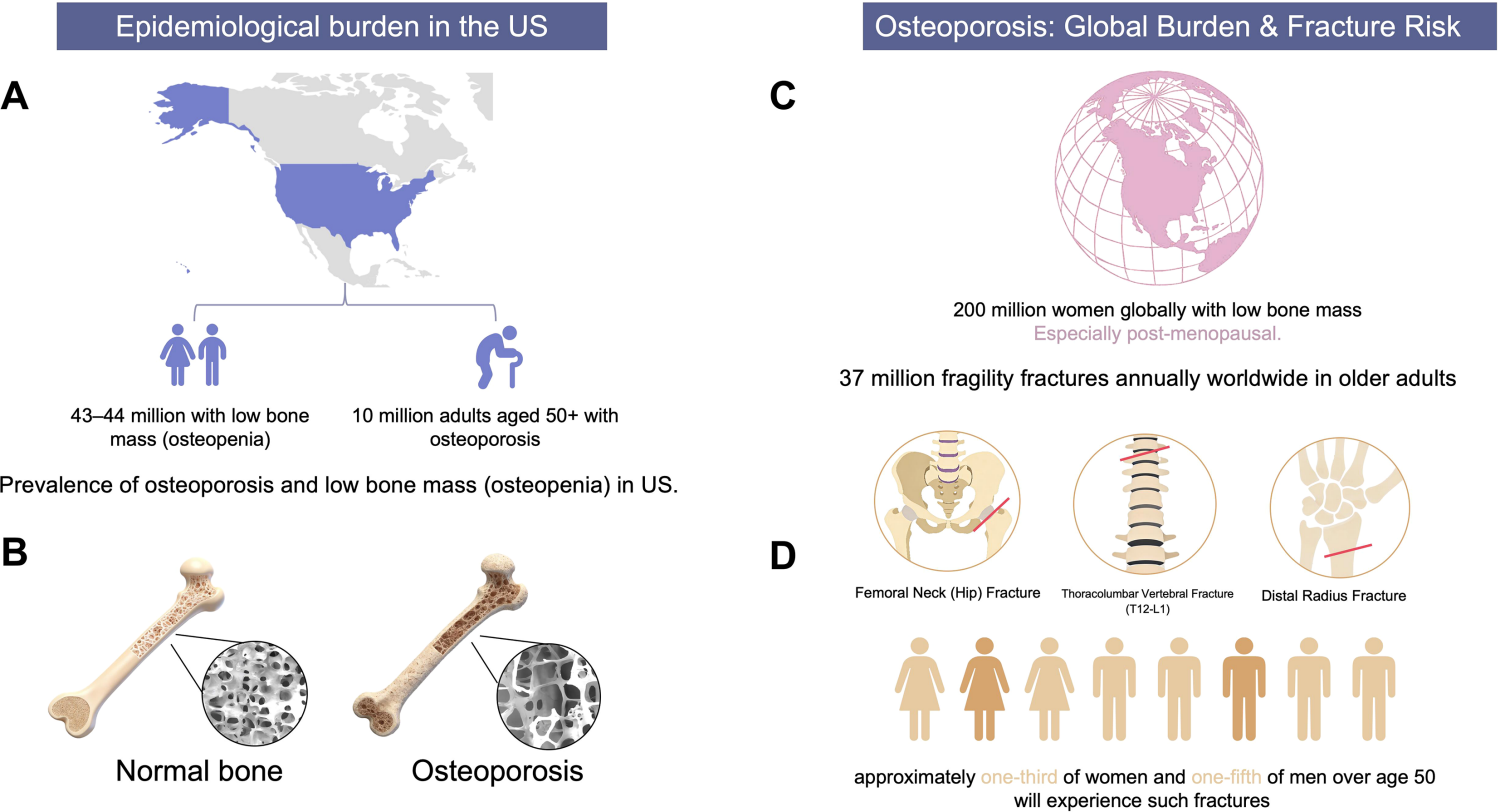
Epidemiological burden and fragility fracture risk of osteoporosis **(A)** Epidemiological burden of osteoporosis in the United States. There are approximately 43–44 million people with low bone mass (osteopenia), and 10 million adults aged 50 years and older living with osteoporosis. **(B)** Global prevalence of low bone mass and annual fragility fracture burden. Globally, 200 million women have low bone mass, with a particularly elevated risk in post-menopausal status; meanwhile, 37 million fragility fractures are reported annually in older adults worldwide. **(C)** Microstructural comparison between normal bone and osteoporotic bone. Osteoporotic bone presents severely damaged trabecular architecture and significant bone loss. **(D)** Major osteoporotic fragility fracture sites and lifetime fracture risk. Three typical fracture locations are marked: femoral neck fracture, thoracolumbar (T12-L1) vertebral fracture, and distal radius fracture. Approximately one-third of women and one-fifth of men over the age of 50 will experience an osteoporotic fragility fracture in their lifetime.

In the United States, approximately 10 million adults aged 50 years and older have osteoporosis, while an additional 43 to 44 million have low bone mass (osteopenia) (4). Globally, low bone mass is estimated to affect 200 million women, particularly following menopause (5). The principal clinical concern is the elevated risk of fragility fractures: studies estimate that up to 37 million fragility fractures occur annually among older adults worldwide, with hip, spinal, and wrist fractures being the most prevalent. The lifetime risk is significant: approximately one-third of women and one-fifth of men over age 50 will experience such fractures (**Figure 1**) (6, 7).

Osteoporotic fractures represent a leading cause of disability and are associated with significantly increased mortality (8). Hip and vertebral fractures, in particular, contribute to substantially higher one-year mortality rates, with excess mortality consistently higher in men than in women (9, 10). Although age-standardized mortality and disability-adjusted life year (DALY) rates related to low bone mineral density have declined modestly in recent decades, the absolute number of deaths attributable to low bone mass has increased. This trend is driven by global population aging and a growing number of high-risk individuals (11).

Despite the availability of evidence-based screening guidelines and effective pharmacotherapies, a persistent treatment gap remains. For example, U.S. data indicate that the proportion of patients receiving osteoporosis medication after hip fracture declined from approximately 40% in 2002 to about 20% by 2011 in certain cohorts, with continued low initiation rates in subsequent years (12). This trend suggests that deficits in osteoporosis management arise not only from limitations in current therapies but also from barriers such as drug accessibility, poor patient adherence, and systemic failures in care transitions (13, 14). Collectively, these findings underscore the urgent need for novel, more effective treatment strategies that address both the underlying pathophysiology and practical barriers to implementation.

Pharmacological management of osteoporosis primarily involves two therapeutic classes: antiresorptive agents and anabolic drugs. Both classes modulate bone remodeling to increase bone mineral density (BMD) and reduce fracture risk. Bisphosphonates, such as alendronate, are the most widely prescribed antiresorptive agents. These compounds bind to hydroxyapatite in the bone matrix and are internalized by osteoclasts, where they inhibit resorptive activity and suppress bone turnover. Denosumab, a fully human monoclonal antibody, targets the receptor activator of nuclear factor kappa-β ligand (RANKL), a key cytokine involved in osteoclast differentiation and activation. Denosumab provides an alternative antiresorptive mechanism by selectively preventing osteoclast formation and function (15–17).

Pharmacological management of osteoporosis primarily involves two therapeutic classes: antiresorptive agents, such as the bisphosphonates and denosumab, as well as the anabolic drugs like teriparatide. While these agents have advanced osteoporosis care, their long-term use presents significant clinical challenges. Prolonged suppression of bone turnover can result in excessively mineralized or “frozen” bone with impaired microdamage repair, increasing the risk of rare but serious complications such as atypical femoral fractures and osteonecrosis of the jaw (18, 19). Teriparatide is limited by preclinical safety concerns which was found to hold osteosarcoma risk in rats, daily subcutaneous administration, and high cost (20–23). These limitations indicate that conventional systemic drug therapy alone may be insufficient to achieve sustained fracture protection across diverse patient populations (24, 25), prompting growing interest in next-generation strategies, including localized delivery systems, combination regimens, and targeted biomaterials (26, 27).

Recent research has expanded its focus beyond bone cells to include the gut as a critical regulatory site (28, 29). The emerging concept of the gut–bone axis is reshaping current understanding of bone homeostasis and osteoporosis pathophysiology. Specifically, the gut–bone axis refers to the bidirectional communication network between the gut microbiota and the skeletal system. Intestinal microorganisms and their metabolites, such as short-chain fatty acids, tryptophan derivatives, and bile acids, which influence bone homeostasis by modulating gut barrier integrity, immune cell polarization like Th17/Treg balance, and systemic levels of estrogen, calcium, vitamin D, and parathyroid hormone. Conversely, bone-derived factors may also affect gut function, although this remains less explored (30, 31). Accumulating evidence indicates that these inter-organ interactions present promising opportunities for developing innovative preventive and therapeutic strategies for osteoporosis (32, 33).

In parallel, advanced biomaterials, including bone cements, hydrogels, and nanostructured materials have demonstrated excellent biocompatibility, tunable mechanical properties, and controllable degradability in bone tissue engineering (34, 35). However, it is important to distinguish between two fundamentally different applications of biomaterials in the context of the gut–bone axis. Local biomaterials, such as bone cements and defect-filling hydrogels, act directly at skeletal sites and do not inherently interact with gut microbiota unless deliberately engineered for systemic immunomodulation. Systemic or gut-targeted biomaterials like oral nanoparticles and probiotic encapsulation systems are designed to pass through or act within the gastrointestinal tract, modulating microbial composition or metabolite production. Most conventional bone biomaterials fall into the first category and, despite their regenerative promise, do not address the systemic metabolic nature of osteoporosis (36, 37). Moreover, they face unresolved challenges including limited long-term efficacy, poor integration with biological pathways, and inability to modulate systemic bone metabolism (26, 27). These limitations raise the question of whether biomaterial platforms could be intentionally designed to target the gut–bone axis, as an approach that remains largely preclinical but offers a distinct therapeutic paradigm.

Several recent reviews have outlined the association between gut microbiota and osteoporosis. However, few targeted critiques and in-depth discussions have focused on the rational design of functional biomaterial platforms—including pH-responsive nanoparticles, probiotic-incorporated hydrogels, and metabolite-releasing scaffolds—to regulate gut-bone crosstalk. To fill this critical research gap, the present review addresses three key objectives. First, it systematically evaluates and stratifies current evidence revealing the mechanistic links between gut microbiota and bone physiology from human, animal and cellular studies. Second, it critically assesses the application of conventional bone biomaterials in osteoporosis treatment and highlights their inherent limitations when used alone. Third, it establishes a comprehensive conceptual framework connecting engineered biomaterials, gut microbiota and bone metabolism, and proposes clear guiding principles to advance future translational research in this emerging field.

## 2 Literature search strategy

PubMed and Web of Science were searched up to March 2026 using combinations of the following keywords: “osteoporosis”, “gut microbiota”, “gut–bone axis”, “short-chain fatty acids”, “tryptophan”, “bile acids”, “probiotics”, “biomaterials”, “bone cement”, “hydrogel”, “nanoparticle”, and “immune modulation”. Only English-language articles were included. Preclinical studies were prioritized if they used ovariectomized, aged, or otherwise osteoporotic animal models. Human studies were included regardless of design but were graded for evidence level (see **Table 1**). Reference lists of retrieved articles were manually screened for additional relevant studies.

**Table 1:** Strength of evidence for gut microbiota–mediated mechanisms in osteoporosis.

Mechanistic pathway	Human evidence	Preclinical *in vivo* evidence	Mechanistic *in vitro* evidence	Key research limitation
SCFAs–GPR43 signaling suppressing osteoclastogenesis	Limited, mainly associative studies linking fecal/serum SCFA levels with BMD and fracture risk.	Strong evidence from OVX and aging-related rodent models with SCFA supplementation showing reduced bone resorption and preserved trabecular bone (38–40)	Strong evidence in osteoclast precursor cultures demonstrating inhibition of NFATc1, TRAF6, and resorptive activity (38, 39)	Lack of large-scale human interventional trials
SCFAs–HDAC inhibition promoting Treg expansion and immune-mediated bone protection	Limited indirect evidence from SCFA profiling and immune phenotype correlations in postmenopausal women.	Strong evidence from murine colitis, OVX, and immune-mediated bone loss models (38, 39)	Strong evidence in T-cell and co-culture systems showing increased FoxP3 expression and IL-10/TGF-β secretion (41, 42)	Human causal evidence remains insufficient
Tryptophan metabolites–AhR signaling regulating gut barrier and bone immunity	No direct clinical trials; mechanistic associations largely absent in humans.	Moderate evidence from OVX mouse studies, particularly Chen et al. (2024), showing IAA/IPA-mediated bone protection via intestinal AhR (43)	Moderate evidence from intestinal epithelial cells and organoid models (43–46)	Human validation and longitudinal biomarker studies lacking
Bile acids–FXR/TGR5 signaling promoting osteogenesis and suppressing resorption	No direct human mechanistic evidence.	Moderate evidence from agonist-treated OVX rodents and bile acid transfer studies (47–50)	Moderate evidence in osteoblast and MSC cultures demonstrating enhanced osteogenic marker expression (47, 48)	Mechanism primarily preclinical with limited translational validation
Probiotic intervention (L. reuteri, LGG, B. longum) preventing bone loss	Preliminary evidence from small-scale RCTs and pilot clinical studies.	Strong evidence across multiple OVX and aged rodent models (51–53)	Moderate evidence from immune–bone co-culture and epithelial barrier models (54–56)	Small sample sizes and heterogeneity of probiotic strains
Estrobolome-mediated estrogen bioavailability via microbial β-glucuronidase	Indirect evidence from metagenomic and hormonal association studies in postmenopausal women (57, 58)	Moderate evidence from GUS inhibitor studies and gnotobiotic models (59–61)	Strong biochemical and enzyme-based mechanistic evidence (59, 62)	Absence of direct clinical intervention studies targeting GUS

In summary, this review offers a focused and critical synthesis that distinguishes itself from existing literature, which primarily focuses solely on either microbiota or biomaterials. By integrating molecular mechanisms, clinical implications, and material innovation, it aims to provide translational guidance for the development of next−generation, interdisciplinary strategies to manage osteoporosis.

## 3 Mechanisms of gut microbiota in osteoporosis

### 3.1 Microbial metabolites

The gut microbiota, primarily comprising the phyla Firmicutes, Bacteroidetes, Actinobacteria, and Proteobacteria, is the largest and most diverse microbial community in the human body. Its metabolic functions are closely associated with host physiology. Recent research indicates that the gut microbiota modulates bone metabolism via the gut–bone axis, exerting significant influence on the initiation, development, and fracture risk in osteoporosis (37, 63). Additionally, gut microbes synthesize numerous bioactive metabolites, including short-chain fatty acids (SCFAs), tryptophan-derived compounds, and bile acid metabolites, which can regulate bone homeostasis by modulating immune responses, inflammatory pathways, and calcium absorption (26, 30, 64).

The following subsections examine these metabolite pathways primarily in the context of postmenopausal osteoporosis driven by estrogen deficiency and age-related bone loss, as these conditions are the most extensively investigated models in the literature. Although inflammatory bone loss, particularly in rheumatoid arthritis, is discussed where relevant, the main focus remains osteoporosis.

As summarized in **Table 1**, SCFA-related pathways currently represent the most robustly supported mechanisms within the gut–bone axis, with convergent evidence from *in vivo*, *in vitro*, and limited human studies. In contrast, mechanisms involving tryptophan metabolites and bile acid signaling remain largely preclinical, highlighting important translational gaps that warrant future longitudinal and interventional studies in human populations.

#### 3.1.1 Short-chain fatty acids

SCFAs are saturated fatty acids containing two to six carbon atoms, formed through microbial fermentation of non-digestible carbohydrates such as dietary fiber and resistant starch. The main SCFAs—acetate (C2), propionate (C3), and butyrate (C4)—account for approximately 90-95% of the total SCFA content, while isobutyrate, valerate, isovalerate, and caproate are produced in smaller amounts (65–67). SCFAs are chiefly synthesized in the colon, where their concentrations decrease from the proximal to distal segments, reaching minimal levels in the terminal ileum (65–67).

SCFA synthesis depends on dietary fiber consumption, microbial community structure, gastrointestinal transit time, and host genetics. In the colon, the acetate:propionate:butyrate molar ratio typically approximates 60:25:15, though this can vary by diet and microbial composition (65–67). Butyrate is the primary energy substrate for colonocytes; acetate and propionate are absorbed into the portal circulation, with propionate largely metabolized by the liver and acetate disseminating systemically (41, 42).

##### 3.1.1.1 SCFAs promote osteogenesis

SCFAs enhance osteogenesis by promoting osteoblast lineage commitment through both direct and indirect mechanisms. Butyrate inhibits histone deacetylase (HDAC), thereby augmenting osteogenic gene transcription. In ovariectomized (OVX) mice, which serve as a model of estrogen deficiency-induced bone loss, butyrate treatment attenuates the loss of trabecular bone, bone volume, and bone mineral density compared with untreated OVX controls (67, 68) This effect has been associated with activation of the Wnt/β-catenin signaling pathway and upregulation of osteogenic markers, including Runx2, Osterix, and alkaline phosphatase (ALP) (40, 67–69).

Propionate has been reported to activate GPR41 and GPR43 in certain cell types, which may stimulate insulin-like growth factor 1 (IGF-1) release and enhance osteoblast function, although the *in vivo* relevance in osteoporotic models requires further validation (70, 71). Propionate has also been shown to facilitate osteogenic differentiation of bone marrow mesenchymal stem cells (BMSCs), collagen synthesis, and mineralized nodule formation (40, 67–69).

In addition to direct actions on bone cells, SCFAs indirectly support bone formation by regulating intestinal calcium transport. For example, SCFAs upregulate the expression of genes involved in calcium absorption, such as Calbindin-D9k, thereby enhancing calcium bioavailability for mineralization (40, 67–69). The effects of SCFAs on bone are dose-dependent. Low concentrations of butyrate promote bone formation, while higher concentrations may inhibit this process, this biphasic response underscores the necessity of precise dosage optimization in therapeutic applications (72). In preclinical studies, SCFAs are commonly administered via drinking water (e.g., butyrate or propionate at 100–200 mM) or intraperitoneal injection (e.g., butyrate at 500 mg/kg/day) (72).

##### 3.1.1.2 SCFAs inhibit osteoclastogenesis

SCFAs inhibit osteoclast differentiation and activity via both immunomodulatory and direct cellular mechanisms. Specifically, SCFAs promote regulatory T cell (Treg) generation and suppress commitment to the Th17 lineage, thereby restoring the RANKL/OPG ratio (73). Notably, butyrate upregulates FoxP3 transcription via HDAC inhibition, thereby enhancing Treg-mediated secretion of IL-10 and TGF-β; these cytokines subsequently suppress osteoclastogenesis (74).

At the cellular level, propionate and butyrate directly inhibit the differentiation of osteoclast precursors and reduce the bone resorption activity of mature osteoclasts. Mechanistically, these SCFAs trigger metabolic reprogramming in osteoclasts by promoting glycolysis and suppressing oxidative phosphorylation, thereby downregulating key differentiation factors such as TRAF6 and NFATc1 (74) (38). Experimental studies demonstrate that SCFA treatment markedly reduces RANKL-induced osteoclast formation and resorption pit area, effects that are significantly attenuated in GPR43-deficient mice, highlighting GPR43 as a critical mediator of SCFA-induced anti-resorptive activity (38).

Furthermore, SCFAs improve intestinal barrier integrity, reducing lipopolysaccharide (LPS) translocation and thereby attenuating LPS/TLR4-mediated inflammatory signaling and osteoclast activation, in OVX models, SCFA supplementation decreases serum levels of proinflammatory cytokines such as TNF-α, IL-6, and IL-1β, which are known to promote osteoclastogenesis in osteoporosis (75, 76).

##### 3.1.3.3 Key molecular targets of SCFAs signaling in bone metabolism

Short-chain fatty acids (SCFAs) regulate bone metabolism through a combination of receptor-dependent and receptor-independent mechanisms, acting on both bone cells and the immune system to maintain skeletal homeostasis (77).

Among receptor-mediated mechanisms, the G-protein-coupled receptors GPR41 (FFAR3) and GPR43 (FFAR2) are expressed in osteoblasts, osteoclast precursors, and immune cells, where they serve as key sensors of SCFAs, including acetate, propionate, and butyrate (70). Although ligand affinities vary across experimental systems, acetate and propionate are generally considered potent activators of GPR43, whereas propionate and butyrate more effectively activate GPR41 (71). Activation of these receptors regulates intracellular calcium dynamics and downstream G protein signaling pathways, thereby modulating inflammatory responses and metabolic processes relevant to bone remodeling (70, 71).

Evidence from *in vivo* studies further suggests that SCFA-mediated bone protection is not exclusively dependent on GPR43 signaling (73). Gpr43^-^/^-^ mice still retain partial protection against bone loss following SCFA supplementation, indicating that additional receptors, particularly GPR41, as well as receptor-independent mechanisms, contribute to the overall skeletal effects (73).

Within bone cells, GPR43 activation has been associated with enhanced osteoblast proliferation and differentiation via PI3K/Akt and ERK1/2 signaling pathways, this is accompanied by increased expression of osteoprotegerin (OPG) and a reduced RANKL/OPG ratio, thereby indirectly suppressing osteoclastogenesis (78, 79). In contrast, GPR41 is highly expressed in sympathetic ganglia and may regulate systemic energy balance through modulation of the sympathetic nervous system, SCFAs acting through GPR41 have been implicated in the regulation of systemic metabolism and potentially the growth hormone–insulin-like growth factor 1 (GH–IGF-1) axis, although direct causal evidence linking this pathway to bone anabolism remains limited (80).

Besides, beyond receptor signaling, SCFAs exert important receptor-independent effects, particularly through epigenetic regulation. Butyrate, in particular, functions as a potent histone deacetylase (HDAC) inhibitor, increasing histone acetylation and promoting Runx2-dependent transcriptional activity, thereby enhancing osteoblast differentiation and bone formation (41). As in immune cells, butyrate promotes the expansion of regulatory T cells, contributing to an anti-inflammatory microenvironment that supports bone preservation and suppresses excessive osteoclastogenesis (81).

Overall, the effects of SCFAs on bone metabolism arise from a multilayered regulatory network that integrates direct and indirect mechanisms. Direct effects include receptor-mediated signaling in osteoblasts and osteoclast precursors, as well as epigenetic regulation via HDAC inhibition and metabolic reprogramming. Indirect effects are primarily mediated through immune modulation, particularly Treg expansion, and improvement of intestinal barrier integrity, which together reduce systemic inflammation and osteoclastogenic signaling (38, 73–76).

#### 3.1.2 Tryptophan metabolites and AhR-mediated regulation of bone immunity

Tryptophan (Trp) is an essential amino acid catabolized through three primary metabolic pathways: the kynurenine (Kyn) pathway, the serotonin (5-HT) pathway, and the indole pathway (82). The Kyn pathway, mediated by indoleamine 2,3-dioxygenases (IDO1/2) and tryptophan 2,3-dioxygenase (TDO), is the predominant route for Trp degradation, yielding Kyn and subsequent metabolites (83). The 5-HT pathway, active mainly in the gastrointestinal tract and central nervous system, produces serotonin and melatonin, while the gut microbiota converts Trp to indole derivatives such as indole-3-acetic acid (IAA), indole-3-propionic acid (IPA), and indole-3-aldehyde (IAld) (84). These microbial metabolites can serve as ligands for the aryl hydrocarbon receptor (AhR), a central transcription factor regulating immune-bone interactions and skeletal homeostasis.

##### 3.1.2.1 AhR signaling in bone metabolism

AhR is a ligand-activated transcription factor that regulates cell differentiation, immune responses, and metabolism, exhibiting context-dependent effects on bone cells.

###### 3.1.2.1.1 Intestinal AhR activation

In an ovariectomy-induced osteoporosis mouse model, Chen et al. showed that OVX mice exhibited altered gut microbiota composition and reduced Trp metabolite levels; supplementation with IAA or IPA restored intestinal barrier integrity and ameliorated bone loss by activating AhR in intestinal epithelial cells. Importantly, in mice with intestinal epithelial-specific AhR deletion (Villin-Cre Ahr^fl/fl), the protective effects of IAA/IPA on both gut barrier and bone were abolished, confirming the essential role of intestinal AhR (43, 85).

###### 3.1.2.1.2 Immune-mediated effects

AhR activation enhances anti-inflammatory IL-10 production from M2 macrophages and promotes Treg differentiation, thereby indirectly suppressing osteoclastogenesis via modulation of the RANKL/OPG axis (46).

###### 3.1.2.1.3 Direct skeletal actions

in osteoblasts, AhR activation can inhibit osteogenesis by upregulating miR-29b-1-5p, thereby suppressing osteogenic gene expression (86). In osteoclasts, the Kyn–AhR pathway modulates NFATc1 and c-Fos expression, whereas microbial IPA inhibits osteoclastogenesis via the PXR/p65 complex (44, 45).

The relationship between kynurenine (Kyn), ROS, and osteoblast differentiation is complex and dose-dependent. In certain osteoblastic cell lines, high Kyn concentrations (e.g., >100 μM) have been reported to increase ROS levels, which paradoxically promoted differentiation under specific oxidative stress conditions, whereas low Kyn concentrations did not (87). However, most studies report that excessive ROS impairs osteogenesis, and thus this pro-osteogenic effect of Kyn-induced ROS should be interpreted with caution and requires independent validation.

Serotonin (5-HT) regulates bone formation in a receptor-dependent and bidirectional manner. Gut-derived 5-HT acting through the HTR1B receptor on osteoblasts inhibits bone formation by suppressing cAMP/PKA-dependent phosphorylation of CREB, thereby reducing osteoblast proliferation (88, 89).

In summary, tryptophan metabolites regulate bone immunity primarily through AhR, but the net effect depends on the specific metabolite, cell type, and disease context. Most evidence comes from rodent models of inflammatory or estrogen-deficient bone loss; human data are lacking. The contribution of direct AhR signaling in osteoblasts versus indirect gut-barrier-mediated effects remains to be dissected.

#### 3.1.3 Bile acids and their derivatives

##### 3.1.3.1 Bone-protective potential in osteoporosis

Alterations in gut microbiota composition and bile acid profiles have been observed in patients with osteoporosis, suggesting that gut microbiota bile acid dysregulation may contribute to bone metabolism disorders (90). Bile acids, synthesized from cholesterol in the liver, play crucial roles not only in lipid digestion and absorption of fat-soluble vitamins but also as signaling molecules regulating energy metabolism, glucose homeostasis, and bone turnover (64, 91). The synthesis and metabolism of bile acids involve enterohepatic circulation and microbial transformation—primary bile acids are converted into secondary bile acids by intestinal microbes, maintaining a dynamic equilibrium within the bile acid pool (92, 93).

Bone remodeling is a continuous process that involves both osteoblast-mediated bone formation and osteoclast-mediated bone resorption. This process depends on a fine balance between these two activities (94, 95). Disruption of this balance in favor of resorption leads to bone loss and structural deterioration, hallmarks of osteoporosis. Bile acids and their receptors, notably farnesoid X receptor (FXR) and Takeda G protein–coupled receptor 5 (TGR5), are expressed in bone cells and play critical roles in bone metabolism (96, 97).

##### 3.1.3.2 FXR in bone metabolism

FXR, a nuclear receptor highly expressed in the liver and intestine, also plays an important role in bone metabolism. FXR is expressed in osteoblasts and osteoclast precursors, and its activation promotes osteoblast differentiation while inhibiting osteoclast formation. In mesenchymal stem cells, activation of FXR enhances Runx2, ALP, and osteocalcin expression, thereby facilitating osteogenic differentiation (47, 48). Conversely, FXR deficiency leads to reduced bone formation and increased osteoclast activity in mice, indicating that FXR is essential for maintaining skeletal homeostasis.

##### 3.1.3.3 TGR5 in bone homeostasis

TGR5, a G protein-coupled receptor expressed in multiple tissues, mediates the bile acid-dependent regulation of bone metabolism. By increasing intracellular cAMP, TGR5 activation suppresses RANKL-induced osteoclastogenesis and resorption. It also attenuates inflammatory cytokine production, thereby alleviating bone loss associated with chronic inflammation (50, 98). In estrogen-deficient induced osteoporosis models, TGR5 expression decreases, whereas pharmacological activation of TGR5 improves bone microarchitecture and density, underscoring its protective function (49).

##### 3.1.3.4 Ursodeoxycholic acid: antioxidant and osteogenic effects

UDCA, a hydrophilic bile acid with potent antioxidant and anti-inflammatory properties, promotes osteogenic differentiation of mesenchymal stem cells (MSCs) while inhibiting adipogenic differentiation. UDCA scavenges reactive oxygen species (ROS), particularly H_2_O_2_, thereby reducing oxidative stress–induced inhibition of osteogenesis. Excessive ROS impairs osteogenic differentiation by upregulating inflammatory cytokines and disrupting MSC function; thus, UDCA exerts significant bone regenerative potential (99).

##### 3.1.3.5 Taurine: bone-protective derivatives

Taurine has demonstrated bone-protective effects. Studies have shown that fecal microbiota transplantation from exercise-conditioned donors increases taurine and UDCA levels, thereby improving bone quality in OVX and aged mice via activation of the apelin signaling pathway and enhancement of gut barrier function (100, 101).

##### 3.1.3.6 Gut microbiota bile acid axis in osteoporosis

The gut microbiota and bile acid metabolism are intricately interconnected. Gut microbes transform primary bile acids into secondary forms, remodeling the bile acid pool composition, while bile acids reciprocally influence microbial structure and function. This bidirectional regulatory network, termed the “gut microbiota bile acid axis”, plays a critical role in the pathogenesis of osteoporosis (102, 103).

Overall, bile acid signaling via FXR and TGR5 shows bone-protective effects in preclinical models, but human evidence is absent. Most data come from OVX rodents or ex vivo cultures. The translation potential of bile acid-targeted therapies for osteoporosis remains to be established.

### 3.2 Mechanisms by which gut microbiota regulate osteoporosis via immune modulation

Gut microbiota regulate through immune modulation, with the Th17/Treg axis occupying a central role in osteoimmunology. Th17 cells promote osteoclast differentiation and activation by producing pro-osteoclastogenic mediators such as IL-17, RANKL, and TNF-α, thereby enhancing bone resorption, whereas Tregs limit osteoclastogenesis by secreting anti-inflammatory cytokines, including IL-10 and TGF-β. Clinical and preclinical studies report a skewed Th17/Treg ratio in osteoporosis and other bone-loss conditions, which contributes to dysregulation of the RANKL/OPG axis and accelerated bone loss (64, 104).

Gut microbes modulate the Th17/Treg balance in a taxon- and context-dependent manner. Segmented filamentous bacteria (SFB) are potent inducers of intestinal Th17 cells and have been implicated in microbiota-dependent models of bone loss, while certain indigenous Clostridium species (clusters IV/XIVa) promote differentiation of inducible colonic Tregs and confer anti-inflammatory, bone-protective effects (105, 106).

Macrophage polarization is another important mediator linking microbiota to bone. Microbial components and metabolites can skew macrophages toward proinflammatory (M1) or anti-inflammatory (M2) phenotypes; however, the direction of polarization depends on bacterial species/strain, host context, and local microenvironment. Akkermansia muciniphila has been reported to modulate macrophage responses through TLR2-dependent pathways in certain models, but its net effect on macrophage polarization (and on bone) is strain- and context-dependent and cannot be universally classified as M1-promoting (107–109).

B cells (Bregs) contribute to osteoimmune regulation. Microbiota and microbiota-derived metabolites can enhance the frequency and IL-10 production of regulatory B cells via FFAR-dependent and HDAC-dependent mechanisms, with downstream involvement of signaling nodes. Conversely, under pathological conditions, B cells can aberrantly express RANKL, thereby directly promoting osteoclastogenesis and local bone erosion (110–112).

In summary, the gut microbiota function as an immunomodulatory and endocrine-like organ, influencing bone remodeling through various context-dependent cellular pathways, including Th17/Treg balance, macrophage polarization, and B cell effector functions. The overall skeletal outcome is determined by the complex interplay among specific microbial taxa, their metabolites, and the host immune status.

#### 3.2.1 Intestinal barrier integrity and systemic immunity

The integrity of the intestinal barrier is critical in preventing excessive immune activation. When the barrier is compromised, bacterial translocation and metabolite leakage can trigger systemic immune responses that, in turn, indirectly affect bone metabolism. Osteoporotic patients often exhibit impaired gut barrier function, leading to the translocation of bacterial components, such as lipopolysaccharide (LPS), into the circulation. This activates the TLR4/NF-κB inflammatory pathway and enhances osteoclastogenesis (75, 113).

Estrogen deficiency increases intestinal permeability, allowing LPS and proinflammatory T cells to migrate from the gut to the bone marrow, skewing the Th17/Treg balance toward osteoclast-promoting differentiation (114, 115). Notably, germ-free or T-cell-deficient mice do not develop bone loss following ovariectomy, underscoring the central role of the microbiota–immune axis in the pathogenesis of osteoporosis (116).

The gut microbiota closely regulates intestinal barrier integrity. Probiotics such as Lactobacillus rhamnosus GG (LGG), Lactobacillus reuteri, and Bifidobacterium longum enhance the expression of tight junction proteins zonula occludens-1 (ZO-1) and occludin, repairing barrier function and reducing LPS leakage (51, 117). Moreover, these probiotics promote Treg-mediated secretion of IL-10 and TGF-β, thereby suppressing osteoclast formation and upregulating OPG expression (51, 56). These findings have shown promise in preclinical models; however, their translational potential remains to be validated in large, controlled human trials, given the strain-specificity and interindividual variability in response.

#### 3.2.2 Inflammaging and aging-related bone loss

Inflammaging, or chronic low-grade inflammation associated with aging, represents a key link between gut barrier dysfunction, immune dysregulation, and bone metabolism. With age, decreased microbial diversity and impaired barrier function lead to sustained systemic inflammation, which in turn activates osteoclasts (118–120). Chang-Jun Li et al. reported that obesity-related dysbiosis induces senescence of bone marrow macrophages, leading to the secretion of granulocyte colony-stimulating factor (G-CSF) and accelerated skeletal degeneration (118). Neutralization of GCA alleviated both obesity- and LPS-induced bone deterioration, highlighting a potential therapeutic target in the gut–immune–bone axis (121).

Aging-related bone loss is increasingly linked to “inflammaging,” driven by gut barrier dysfunction and microbial dysbiosis. However, most studies are correlational, and causal interventions targeting inflammaging to improve bone health in aged humans have not yet been demonstrated (118–120).

In summary, the gut microbiota influences bone via immune pathways centered on the Th17/Treg balance, macrophage polarization, and B-cell functions. The strength of evidence varies: Th17/Treg regulation by specific bacterial taxa (e.g., SFB, Clostridium) is robust in animal models, but human data are correlational. Macrophage polarization by microbiota remains largely inferential. The translational potential of probiotic interventions requires rigorous human trials.

### 3.3 Hormonal and nutritional pathways linking gut microbiota to bone health

Bone health is regulated by several hormones, such as estrogen and parathyroid hormone (PTH), and by essential nutrients, including calcium, phosphorus, and vitamin D, the gut microbiota influences bone metabolism through three principal mechanisms: (i) direct metabolism of steroid hormones (e.g., estrogen) affecting their bioavailability; (ii) regulation of intestinal absorption of minerals, particularly calcium and phosphorus; and (iii) modulation of vitamin D metabolism and signaling. These pathways are highly interconnected, forming a complex regulatory network that sustains skeletal homeostasis (58, 122, 123).

#### 3.3.1 Bidirectional regulation between gut microbiota and estrogen and its impact on bone health

##### 3.3.1.1 Microbial and probiotic modulation of estrogen bioavailability

Microbial enzymatic regulation of estrogen biotransformation represents a major mechanism by which the gut microbiota modulates systemic estrogen availability and, consequently, bone homeostasis (124). Estrogens exert antiresorptive and proanabolic effects on the skeleton by inhibiting osteoclast differentiation and promoting osteoblast function (125). Gut bacteria participate in enterohepatic and peripheral steroid metabolism via an enzymatic repertoire that includes β-glucuronidases and other deconjugating enzymes, thereby altering circulating and tissue levels of bioactive estrogens (62).

A canonical microbial pathway for estrogen reactivation is mediated by gut microbial β-glucuronidases (GUS), which hydrolyze hepatic glucuronide conjugates to liberate free estrogens for intestinal reabsorption; this estrobolome activity has been demonstrated *in vitro* and *in vivo* and is widely accepted as a determinant of host estrogen exposure (59). Recent metagenomic and biochemical studies have expanded the catalog of steroid-transforming enzymes encoded by human gut bacteria, identifying enzyme families such as Δ^4^;-3-ketosteroid 5β-reductases, 3β-hydroxysteroid dehydrogenase/Δ^54^; isomerases, and Δ^6^-3-ketosteroid reductases; these reductases can catalyze multi-step conversions of steroid precursors and are broadly prevalent in human gut metagenomes, with evidence of female enrichment in some cohorts (57, 126).

Conversely, specific gut bacteria can inactivate potent estrogens: for example, a gut-derived 3β-HSD encoded by Klebsiella spp. catalyzes estradiol degradation, and colonization or gavage with such strains in animal models lowers systemic estradiol and produces relevant behavioural and physiological phenotypes, linking microbially mediated E2 catabolism to host endocrine and neuropsychological outcomes (60).

Microbiota can also indirectly affect peripheral estrogen biosynthesis: probiotic or community-level modulation of the gut environment has been reported to increase peripheral steroidogenic enzyme expression (e.g., aromatase) and to raise circulating estradiol in ovariectomized rodent models, indicating a route by which microbiota composition alters host steroidogenesis (61, 127).

In summary, these lines of evidence support a bidirectional regulatory network in which microbial GUS activity reactivates conjugated estrogens while microbial reductases and dehydrogenases catabolize bioactive steroids; a balanced microbiota thus helps sustain adequate bioactive estrogen levels and skeletal stability, whereas dysbiosis may perturb these enzymatic activities, reduce estrogen availability, and contribute to estrogen-deficiency-related bone loss.

##### 3.3.1.2 Probiotic effects independent of estrogen synthesis

Independent of effects on steroidogenesis, specific probiotics exert bone-protective actions through immunomodulation and direct regulation of bone cell signaling. For example, LGG has been shown to attenuate osteoclastogenesis in ovariectomized rodent models, in part by suppressing activation of the NLRP3 inflammasome and downstream caspase-1/IL-1β signaling, concomitant with improvements in trabecular microarchitecture and reductions in serum bone resorption markers (52, 54, 128). Several Lactobacillus plantarum strains inhibit osteoclast differentiation and activity by downregulating RANKL expression or modulating the RANKL/RANK/OPG axis in preclinical models (55, 129, 130). Mixed-strain formulations may produce additive or synergistic effects (131, 132). The bone-protective actions of L. reuteri (e.g., strain ATCC PTA 6475) reduce intestinal and systemic inflammation and prevent estrogen-deficiency-associated bone loss in rodents, providing mechanistic plausibility for translational investigation in humans (51, 133).

Overall, the gut microbiota influences estrogen-related bone health through two distinct mechanisms: (i) enzymatic modification of estrogen bioavailability (estrobolome), supported by *in vitro* and animal studies but lacking human causality; (ii) immunomodulatory actions independent of estrogen, which are better documented in ovariectomized models. Future research should dissect whether estrogen-dependent and -independent pathways are additive or synergistic.

#### 3.3.2 Regulation of calcium and phosphorus absorption by gut microbiota

Calcium (Ca) and phosphorus (P) are essential minerals for maintaining bone mass and structural integrity. Their intestinal absorption and systemic utilization are tightly regulated by hormonal, nutritional, and microbial factors (134–136).

The gut microbiota profoundly influences calcium and phosphorus bioavailability through several mechanisms. The evidence derives from three levels:

##### 3.3.2.1 General intestinal physiology

SCFAs (acetate, propionate, butyrate) lower luminal pH and upregulate calcium transporters such as TRPV6 and PMCA1b, enhancing mineral absorption (137–143).

##### 3.3.2.2 Osteoporosis-specific animal models

OVX mice show reduced SCFAs and impaired calcium absorption; butyrate supplementation partially restores these parameters (141, 142).

##### 3.3.2.3 Probiotic intervention studies

Lactobacillus casei and Bifidobacterium longum have been reported to increase intestinal NaPi-IIb expression, improving phosphate absorption and bone mineralization in rodents, but human data are scarce (144, 145).

Additionally, the gut microbiota influences systemic mineral metabolism through indirect endocrine effects (146, 147). Butyrate produced by the intestinal microbiota is required for the anabolic action of intermittent parathyroid hormone (iPTH) in mice: depletion of the microbiota abolishes PTH-induced bone formation, while restoration of physiologic butyrate levels rescues it. Mechanistically, butyrate promotes the expansion of bone-marrow Tregs via signaling through GPR43 in dendritic cells and a GPR43-independent pathway in T cells; these Tregs then stimulate CD8 T cells to produce the osteogenic Wnt ligand Wnt10b, which drives Wnt-dependent bone formation (41).

Overall, while SCFAs clearly enhance mineral absorption in healthy intestines, direct evidence that microbiota-targeted interventions improve calcium/phosphorus status specifically in osteoporotic humans is lacking. Most studies are performed in non-osteoporotic rodents or ex vivo systems. Translating these findings requires controlled trials in osteoporotic populations with baseline dysbiosis.

#### 3.3.3 Interactions between gut microbiota and vitamin D metabolism

Vitamin D, a critical regulator of calcium and phosphorus homeostasis, exerts many of its biological effects through the vitamin D receptor (VDR), a nuclear transcription factor widely expressed in tissues such as the intestine, kidney, and bone. Recent evidence suggests that the gut microbiota and vitamin D/VDR signaling engage in a bidirectional regulatory axis, in which each component influences the function of the other, ultimately affecting mineral metabolism and bone health (148).

On the one hand, vitamin D/VDR signaling shapes gut microbial composition and function. Activation of VDR strengthens the intestinal barrier by upregulating tight junction proteins, thereby reducing permeability and local inflammation (148, 149). VDR also regulates innate antimicrobial defenses; for example, it controls the expression of Paneth-cell α-defensins and other antimicrobial peptides, thereby maintaining intestinal microbial homeostasis (150, 151). In animal models, VDR deficiency (or vitamin D deficiency) leads to marked dysbiosis, including reduced levels of beneficial bacteria such as Akkermansia muciniphila, impaired defensin production, gut barrier collapse, and systemic inflammation (150, 152).

On the other hand, the gut microbiota modulates vitamin D metabolism, signaling, and bioavailability via multiple mechanisms. Microbial metabolites can enhance intestinal VDR expression and activity. Butyrate, acting as an HDAC inhibitor, promotes chromatin accessibility and transcriptional activation of VDR-target genes (153, 154).

Additionally, certain bile acid derivatives, notably lithocholic acid (LCA), function as endogenous VDR ligands, further activating VDR-mediated signaling (155, 156).

Gut microbes may also influence the enzymatic steps of vitamin D activation. Although the data are still emerging, some studies suggest that microbial metabolites can regulate the expression of vitamin D hydroxylases (e.g., CYP2R1 in the liver, CYP27B1 in the kidney), thereby potentially promoting conversion to the bioactive form, 1,25-dihydroxyvitamin D. Conversely, dysbiosis and associated inflammation may suppress CYP27B1 activity, impairing vitamin D activation and downstream signaling (148, 152, 157).

Clinical and translational evidence also supports this interaction between microbiota and vitamin D. In postmenopausal women, lower serum 25-hydroxyvitamin D [25(OH)D] levels correlate with reduced gut microbial diversity, while higher 25(OH)D is associated with more favorable microbial taxa (158–160). Some probiotic interventions have been reported to increase VDR expression in intestinal epithelium and modestly raise circulating 25(OH)D levels, suggesting that modulation of the microbiota can influence vitamin D status (161, 162).

In summary, vitamin D/VDR signaling and the gut microbiota exhibit a mutually reinforcing relationship. Vitamin D maintains intestinal barrier integrity and microbial balance, whereas microbial metabolites enhance VDR activation and vitamin D metabolism. Disruption of this axis, whether from dysbiosis or vitamin D deficiency, impairs mineral homeostasis, promotes inflammation, and contributes to bone loss, underscoring its importance in osteoporosis prevention and therapy.

**Figure 2:**
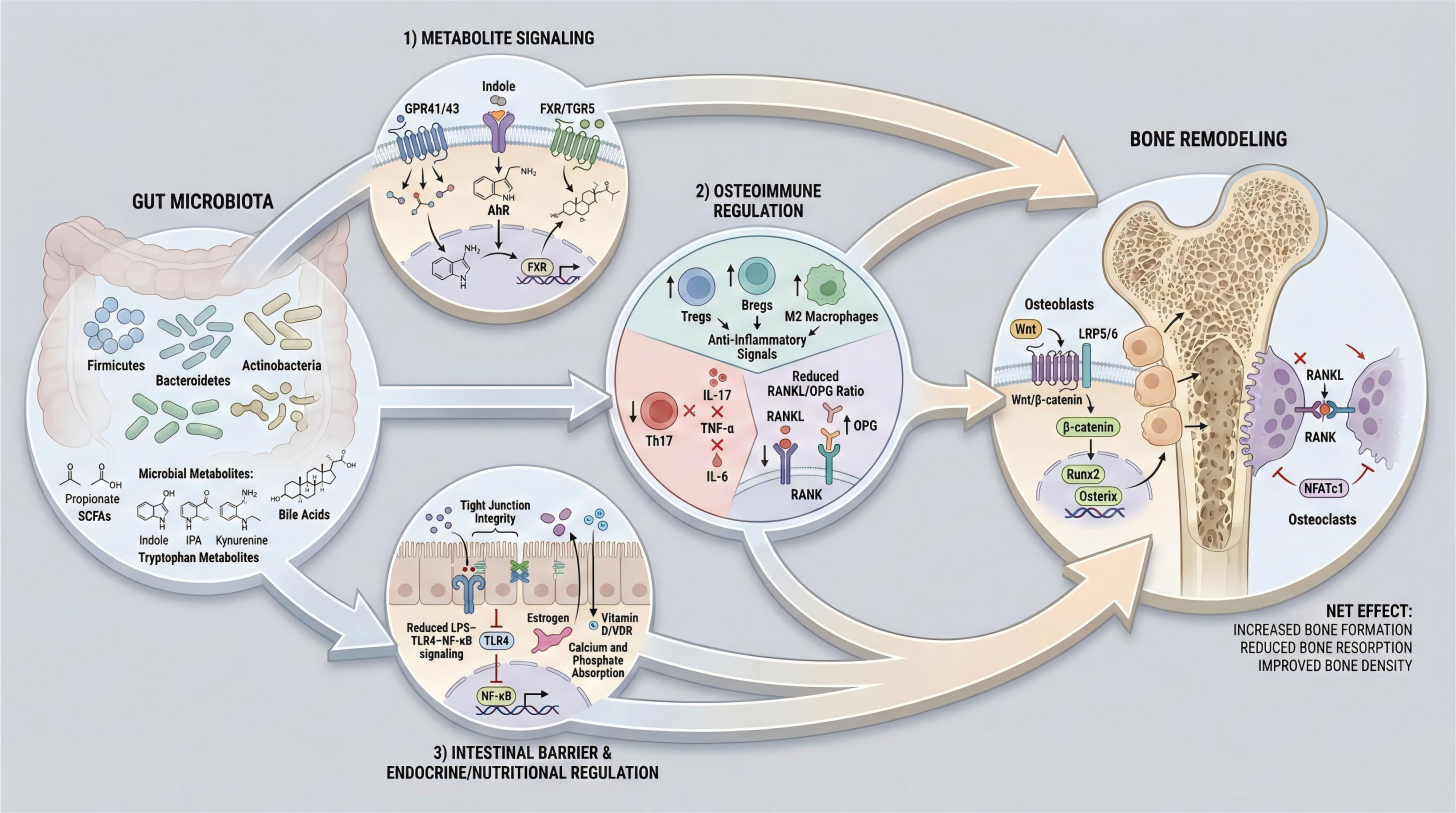
Gut microbiota-driven modulation of bone remodeling.

A schematic overview of the multifaceted mechanisms by which gut microbiota orchestrates bone metabolism in osteoporosis is presented in **Figure 2**.

Gut microbiota modulates osteoporosis pathogenesis through three major interconnected signaling axes, including microbial metabolite regulation via short-chain fatty acids, tryptophan derivatives and bile acid metabolites; osteoimmune modulation involving the Th17/Treg balance, macrophage polarization and intestinal barrier integrity; as well as hormonal and nutritional control of estrogen activity, calcium/phosphorus absorption and vitamin D/VDR signaling, thereby coordinately regulating bone formation and resorption to maintain skeletal homeostasis.

## 4 Emerging biomaterials targeting gut microbiota for osteoporotic bone repair

The gut microbiota plays a pivotal role in the pathogenesis of osteoporosis through multiple pathways, including the regulation of systemic inflammation, modulation of calcium absorption, and orchestration of gut–bone axis crosstalk. Osteoporotic bone loss and associated osteoporotic bone defects have emerged as prevalent and intractable clinical challenges, characterized by impaired bone metabolism, insufficient osteogenic capacity, persistent low-grade inflammation, and elevated fracture risk. All these features are closely associated with gut microbiota dysbiosis and significantly compromise patients’ long-term quality of life. Autologous bone grafting, the classical strategy for bone repair, is severely limited in osteoporotic bone reconstruction by insufficient donor bone mass, donor site morbidity, and its inability to target gut microbiota dysbiosis, a key driver of osteoporotic progression (163–167). Emerging biomaterials that directly link local osteoporotic bone repair to gut microbiota regulation via the gut–bone axis address the unmet clinical need for therapeutic strategies that simultaneously target local bone defects and systemic gut microbiota imbalance—an advantage not offered by conventional bone repair strategies.

Synthetic bone substitute biomaterials have been developed to address the limitations of autologous grafts; however, conventional porous bioceramics and inert non-degradable materials fail to adapt to the disordered immune microenvironment, abnormal bone turnover, and gut microbiota dysbiosis inherent to osteoporosis (168–171). Unlike traumatic or tumor-derived bone defects, osteoporotic bone injury is driven by systemic metabolic disturbance and gut microbiota imbalance, necessitating biomaterials that extend beyond passive structural support to actively regulate both local bone repair and the gut–bone axis. Existing conventional biomaterials lack such dual regulatory capacity, rendering them inadequate for osteoporotic bone repair. Gut microbiota-based strategies are therefore essential to complement advanced biomaterials by targeting the systemic root cause of osteoporosis, thereby enhancing repair efficacy and preventing disease progression.

Advanced functional biomaterials with customizable degradation characteristics and multi-dimensional biological regulatory functions are uniquely positioned to bridge this gap. Beyond local bone defect filling, these biomaterials exert comprehensive regulatory effects on local immune responses, angiogenesis, and osteogenic metabolism, while indirectly regulating systemic inflammatory homeostasis and intestinal microbial balance via the gut–bone axis (171–173). This cross-tissue regulatory loop constitutes a core mechanistic foundation, enabling biomaterials to simultaneously target localized osteoporotic bone defects and systemic gut microbiota dysbiosis, thereby achieving synergistic therapeutic effects unattainable via conventional biomaterials that merely provide local tissue repair.

**Figure 3:**
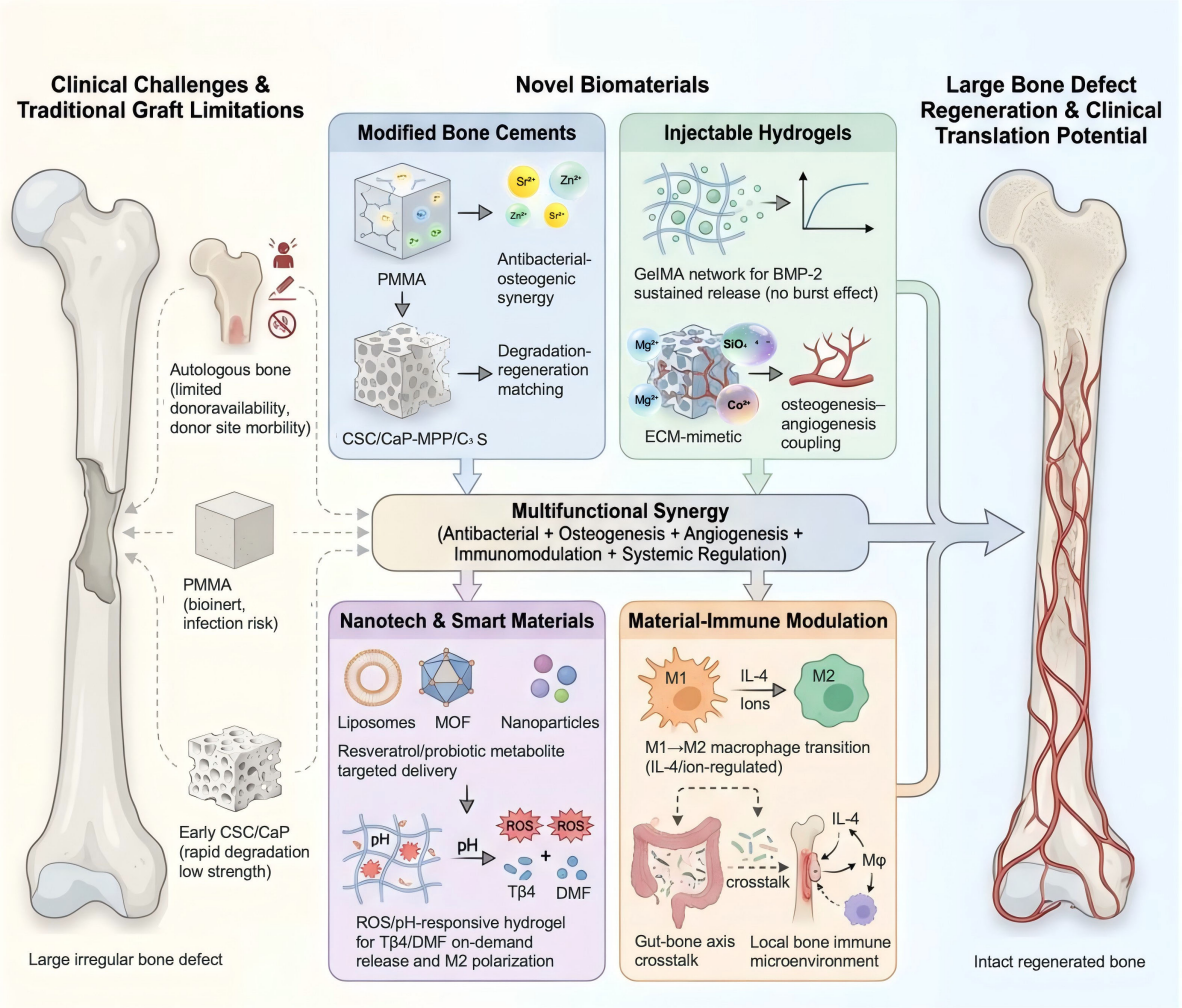
Multifunctional biomaterial strategies targeting gut microbiota for osteoporotic bone defect regeneration.

**Figure 3** systematically illustrates the core clinical obstacles in osteoporotic bone repair, which are linked to gut microbiota dysbiosis, the inherent limitations of conventional bone grafting strategies (failure to target the gut–bone axis), and novel multifunctional biomaterial-oriented therapeutic pathways that directly connect local bone repair to gut microbiota regulation. By synergistically regulating local immune polarization, vascular remodeling, and osteogenic differentiation while coordinating systemic gut microbial metabolism, these multifunctional biomaterials achieve targeted repair of osteoporotic bone defects and reverse gut microbiota dysbiosis to further alleviate osteoporotic bone loss.

Conventional bone repair strategies, such as autologous grafts and PMMA, cannot reverse gut microbiota dysbiosis, a core pathological factor driving osteoporosis progression, and exhibit poor adaptability to the chronic inflammatory and metabolic disorder microenvironment of osteoporotic bone. Multifunctional modified bone cements, injectable hydrogels, intelligent nanodelivery systems and immune-regulatory biomaterials are rationally designed to integrate local bone defect repair with systemic gut microbiota homeostasis through the gut–bone axis. Local biomaterial interventions modulate systemic immune responses and intestinal microecology, thereby facilitating vascularized bone regeneration under osteoporotic conditions. This dual-targeted therapeutic strategy compensates for the deficiencies of traditional bone repair approaches and offers promising translational candidates for comprehensive osteoporosis treatment.

### 4.1 Modified bone cements and implant materials: osteoporotic-focused tools with gut–bone axis regulation

Bone cements and implantable materials designed for osteoporotic bone repair—distinct from those for general orthopedic applications—fulfill dual roles: they act as local repair tools for osteoporotic fractures and as mediators of gut–bone axis regulation to target the systemic etiology of osteoporosis. Conventional bone cements, designed for traumatic defect repair or prosthetic fixation, are incompatible with the osteoporotic microenvironment (174–176). In contrast, modified materials optimized to match osteoporotic bone metabolism and target gut microbiota via systemic metabolic regulation address the unmet need for therapeutic strategies that link local repair to systemic gut microbiota balance.

#### 4.1.1 Biodegradable alternatives: dynamic scaffolds synchronizing with osteoporotic bone regeneration and gut microbiota regulation

Traditional polymethyl methacrylate (PMMA) bone cement is a non-degradable implant material with inherent biological inertness that limits bone integration and exacerbates local chronic inflammation (177–180). This is a critical concern for osteoporotic individuals, as chronic inflammation accelerates osteoclast activation and bone loss, forming a vicious cycle linked to gut microbiota dysbiosis. Notably, the reliance of PMMA on antibiotics for anti-infection intervention disrupts local microecology and indirectly compromises intestinal microbial homeostasis through the gut–bone axis (181), thereby exacerbating osteoporotic bone metabolism. Such drawbacks directly link the inherent limitations of PMMA to gut microbiota regulation, distinguishing this mechanism from conventional discussions focused solely on infection prevention.

In contrast, biodegradable bone cements (calcium sulfate, calcium phosphate) exhibit progressive degradation, enabling replacement of the material by newly formed bone tissue and aligning with the physiological remodeling demands of osteoporotic bone (182–184). However, traditional single-component degradable cements lack the capacity to regulate the gut–bone axis, limiting their efficacy in gut microbiota-associated osteoporosis. Composite modification strategies address this gap by optimizing degradation behavior, mechanical properties, and biological functions to synchronize material degradation with osteoporotic bone healing and enable targeted gut–bone axis regulation (not solely focusing on material performance).

For instance, the incorporation of magnesium polyphosphate (MPP) and tricalcium silicate (C_3_S) into a CSC-based matrix optimizes setting time and compressive strength, providing structural support for osteoporotic defects (185, 186). Meanwhile, strontium, a bioactive anti-osteoporotic ion, incorporated into these cements directly links local material performance to systemic gut microbiota regulation: strontium chelate combined with plant polysaccharides promotes bone regeneration via the gut-liver-bone axis (187), providing a concrete example of how these modified cements target both local repair and gut microbiota balance.

Loading biodegradable bone cements with prebiotic components (rather than merely general bioactive factors) further enhances their relevance to the gut–bone axis. Prebiotics delivered locally enter the systemic circulation to regulate intestinal flora composition, improving calcium absorption and bone metabolism via the gut–bone axis—critical for reversing gut microbiota dysbiosis in osteoporotic patients (36). Importantly, the local anti-inflammatory effects of these cements reduce systemic chronic inflammation, maintaining intestinal barrier integrity, stabilizing beneficial gut microbiota (e.g., Bifidobacterium, Lactobacillus), and decreasing pro-inflammatory cytokines that drive bone loss and gut dysbiosis (188, 189). This forms a positive regulatory loop between local repair and gut microbiota homeostasis, reflecting biological synchrony between material degradation, bone regeneration, and gut microbiota regulation (not merely material performance).

### 4.2 Injectable hydrogels and scaffolds: bridges between gut microbiota regulation and osteoporotic bone repair

Injectable hydrogels associated with the gut microbiota–osteoporosis axis differ substantially from conventional hydrogels designed merely for local defect filling. They are specifically engineered as functional platforms for gut-targeted delivery, probiotic metabolite transportation, and gut–bone axis-related immune modulation. Rather than providing a general overview of hydrogel applications, this review highlights the unique advantages of hydrogels, including ECM mimicry, minimally invasive administration, and controllable drug release. These inherent properties make hydrogels uniquely suited to bridge gut microbiota regulation and osteoporosis treatment, and define the core research focus of the present discussion.

Injectable hydrogels mimic the native ECM, supporting mesenchymal stem cell proliferation and osteogenic differentiation—critical for osteoporotic bone with impaired cellular activity (190–193). Their three-dimensional network enables sustained delivery of gut-relevant bioactive agents (prebiotics, probiotic metabolites) and anti-osteoporotic molecules, linking local repair to systemic gut microbiota regulation (193–195). Injectable composite scaffolds incorporating hydroxyapatite and bioglass enhance osteoconductivity while delivering bioactive ions (magnesium, strontium, selenium) that regulate the gut–bone axis and restore gut microbiota balance (196–199).

#### 4.2.1 Controlled drug delivery: targeting osteoporotic bone repair and gut microbiota homeostasis

The unique controlled release performance of hydrogels meets two key demands: long-term bone metabolism regulation in osteoporosis, and sustained systemic delivery of prebiotics and probiotic metabolites for gut microbiota modulation.

Representative research advances closely aligned with the central theme include the integration of resveratrol—a natural polyphenol with anti-osteoporotic and gut-regulating functions—into injectable hydrogels for prolonged local release. Resveratrol exerts synergistic effects with gut microbiota to ameliorate bone metabolism. Hydrogel-based delivery further modulates the gut–bone axis, thereby facilitating bone repair in osteoporotic lesions and reconstructing intestinal microecological homeostasis (200–204). Another example is colon-targeted postbiotic delivery via hydrogels: postbiotic nanoparticles delivered locally enter the systemic circulation to regulate intestinal microbial composition and systemic inflammation, alleviating osteoporosis via the gut–bone axis (205). This establishes a direct correlation between hydrogel technology and the central research theme, featuring examples that are neither overly generalized nor futuristic, but rather concrete demonstrations of hydrogels acting as a bridge between gut microbiota regulation and osteoporotic bone repair.

#### 4.2.2 Biomimetic porous structures: promoting angiogenesis in osteoporotic bone with gut microbiota regulation

Angiogenic hydrogel systems discussed herein are specifically designed to address osteoporotic fracture healing and poor bone quality (not general tissue regeneration), with a clear link to gut microbiota regulation. Osteoporotic bone is characterized by impaired vascularization, which exacerbates bone loss and delays repair; the porous structure of hydrogels promotes endothelial cell infiltration and capillary sprouting, while their capacity to deliver bioactive ions links angiogenesis to gut–bone axis regulation.

Concrete osteoporosis-relevant examples include a dual-crosslinked magnesium-ion-containing hydrogel (GelMA/TCS/POSS-Mg), which promoted angiogenesis and osteogenesis in a rat calvarial defect model of osteoporosis (206–208). Magnesium ions not only enhance local angiogenesis but also regulate intestinal microbial homeostasis via the systemic circulation, improving bone metabolism and reversing gut dysbiosis (209, 210). Hydrogels incorporated with organic selenium can ameliorate osteoporosis via regulating gut microbiota composition and fecal metabolic profiles, and exert synergistic modulation on local angiogenesis, osteogenesis and the systemic gut–bone axis (199). Such research evidence directly links angiogenic hydrogels to gut microbiota regulation in the treatment of osteoporosis. Different from conventional studies adopting simple calvarial defect models, these investigations focus specifically on osteoporotic pathological conditions, which further highlights their practical significance and research pertinence.

### 4.3 Nanotechnology and smart materials: targeted gut–bone axis regulation for osteoporosis

The therapeutic routes of nanomaterials involve distinguishing among oral gut targeting, systemic circulation, and local bone delivery (avoiding loose merging of distinct strategies). The unique advantage of nanotechnology lies in its ability to enable precise delivery of gut microbiota-modulating agents (probiotics, postbiotics) while supporting local osteoporotic bone repair—highly relevant to the central research focus. Unlike general nanomaterial reviews, this discussion focuses on nanosystems that directly link gut targeting to osteoporotic bone repair.

#### 4.3.1 Nanoscale carrier systems: cross-scale delivery for gut–bone axis regulation

Probiotics regulate osteoporotic bone metabolism by increasing calcium absorption and modulating inflammatory cytokines (e.g., IFN-γ) (211, 212); however, free oral probiotics exhibit poor stability and bioavailability, a gap addressed by nanocarriers.

Concrete, route-specific examples include orally administered degradable nanoarmor-assisted probiotics (designed for gut targeting), which remodel the gut microenvironment and treat osteoporosis by regulating intestinal flora and reducing systemic inflammation (213)—a direct gut-targeted strategy.

In contrast, resveratrol-encapsulated polymeric nanoparticles are designed for dual local bone and gut delivery, improving stability and enabling sustained release at both sites to enhance osteoblastic differentiation and gut microbial regulation (202, 203). Colon-targeted engineered postbiotics nanoparticles (systemic delivery) alleviate osteoporosis via the gut–bone axis, precisely regulating the gut microenvironment and systemic bone metabolism (205). These examples clearly define the therapeutic route, linking nanocarriers to both gut microbiota and osteoporotic bone repair.

#### 4.3.2 Environment-responsive materials: precision therapy for gut–bone axis regulation in osteoporosis

Environment-responsive materials discussed herein link local osteoporotic defect signals (chronic inflammation, oxidative stress) to systemic gut microbiota regulation, avoiding discussions on general implant microenvironments. These “smart” materials trigger on-demand release of anti-inflammatory agents or gut-modulating molecules, converting pathological signals into therapeutic actions while reducing systemic inflammation to restore gut microbiota balance.

A representative example is an ROS-responsive injectable hydrogel fabricated from hyaluronic acid and carboxymethyl chitosan, which releases anti-inflammatory agents as dynamic hydrogel bonds dissociate under oxidative stress—a hallmark of osteoporotic bone defects (214). This reduces local inflammation, which in turn lowers the systemic inflammatory burden—maintaining intestinal barrier integrity and stabilizing gut microbial composition, critical for reversing gut dysbiosis in osteoporotic patients (188, 189). A second example involves a hydrogel containing black phosphorus nanosheets and palladium nanozymes, which scavenges ROS, induces M2 macrophage polarization, and enhances angiogenesis/bone formation, while modulating systemic inflammation to regulate the gut–bone axis and restore gut balance (215).

### 4.4 Material–immune modulation: linking local osteoporotic immune response to gut microbiota homeostasis

This section focuses on gut microbiota-driven immune mechanisms in osteoporosis, clearly distinguishing between local material-induced macrophage responses at the scaffold interface and systemic immune regulation via the gut–bone axis, while rigorously connecting these two levels. Unlike conventional osteoimmunology summaries, this section emphasizes the interaction between material-mediated immune modulation and gut microbiota-derived immune signals in enhancing osteoporotic bone repair.

Macrophages serve as central regulators: material properties (surface topography, ion release) influence polarization toward pro-regenerative M2 phenotypes, which suppress chronic inflammation—a key factor in osteoporotic bone loss linked to gut microbiota dysbiosis (209, 216–219). Material-mediated M2 polarization reduces systemic inflammation, maintaining gut microbial stability and improving bone metabolism via the gut–bone axis (181, 188), forming a bidirectional crosstalk loop between local immune response, material intervention, and gut microbiota.

#### 4.4.1 Macrophage polarization: osteoporosis-specific immune regulation linked to gut microbiota

Concrete osteoporosis-relevant examples include IL-4 delivery via hydrogels, which steers macrophage polarization toward M2 phenotypes—accelerating bone regeneration in aged rat cranial defects (a model of age-related osteoporosis) by inhibiting the NLRP3 inflammasome in macrophages (220). This M2 polarization also leads to the secretion of anti-inflammatory cytokines (e.g., IL-10) that reduce systemic inflammation, restoring gut microbiota balance—directly linking local immune modulation to gut microbiota regulation. M2 macrophages also secrete IL-10, which promotes osteogenic differentiation of MSCs and reduces pro-inflammatory cytokines that drive gut dysbiosi (221), further reinforcing gut–bone-immune crosstalk. These examples clarify that the discussion focuses on mechanisms specifically relevant to osteoporotic bone, not general regenerative immunology.

#### 4.4.2 Correlation with the gut–bone axis: evidence-based integration of material immune modulation and gut microbiota regulation

Concrete evidence includes the observation that Bacillus coagulans (a probiotic) ameliorates inflammatory bone loss in post-menopausal osteoporosis via the “Gut-Immune-Bone” axis (181). Material-mediated immune modulation (e.g., M2 polarization) enhances this effect by reducing systemic inflammation, creating a synergistic loop between local immune response and gut microbiota. Organic selenium compounds (delivered via hydrogels or nanocarriers) modulate gut microbiota composition and fecal metabolite profiles, reducing systemic inflammation and enhancing material-induced M2 polarization (199)—directly linking gut microbiota signals to local immune modulation. Magnesium-containing biomaterials promote M2 polarization, reduce systemic inflammation, and maintain gut microbial stability (209), with experimental data demonstrating improved bone density and gut microbiota balance in osteoporotic models. These examples provide critical evidence for the integrative framework, illustrating how local material immune modulation interacts with gut microbiota to enhance osteoporotic bone repair.

### 4.5 Summary and future perspectives

The core advancement of these novel biomaterials lies in their transition from passive structural support to active multi-dimensional regulation, integrating local osteogenic, angiogenic, and immunomodulatory functions with systemic gut–bone axis regulation and gut microbiota homeostasis.

To systematically clarify the translational logic of biomaterial strategies targeting gut microbiota and osteoporotic bone repair, the key characteristics of different biomaterial categories are summarized as follows. Modified biodegradable bone cements adopt an osteoporosis-oriented local therapy combined with gut regulation strategy, with key functions including dynamic mechanical support, ion release (Sr²^+^, Mg²^+^), and prebiotic delivery. Their connection to the gut–bone axis lies in ion and prebiotic delivery that modulates gut microbiota, as well as anti-inflammatory effects that stabilize the gut barrier, and their relevance to osteoporosis is reflected in matching osteoporotic bone metabolism and addressing chronic inflammation and bone loss. Injectable hydrogels also adopt an osteoporosis-oriented local therapy combined with gut regulation strategy, featuring ECM mimicry, controlled release of resveratrol and postbiotics, and angiogenesis promotion. They link to the gut–bone axis through postbiotic and resveratrol delivery that regulates gut microbiota and ion release that modulates the gut–bone axis, and are suitable for osteoporotic bone due to their minimally invasive nature and ability to support impaired osteogenic cells. Nanoscale carrier systems employ a gut-targeted plus local bone delivery strategy, with key functions of probiotic/postbiotic delivery, targeted drug release, and enhanced bioavailability; their gut–bone axis connection is achieved through oral nanoarmor targeting the gut and systemic delivery linking gut modulation to bone repair, and they address osteoporosis by overcoming probiotic instability and enabling precision regulation of the gut–bone axis. Immuno-active biomaterials adopt an integrative gut–bone strategy, focusing on M2 macrophage polarization, anti-inflammatory effects, and immune modulation; they connect to the gut–bone axis by reducing systemic inflammation through local immune regulation and restoring gut microbiota balance, and target the chronic inflammation linked to osteoporotic bone loss and gut dysbiosis. In contrast, conventional biomaterials (PMMA, single-component CaP) only perform local bone repair, with key functions limited to structural support and low bioactivity; they have no direct connection to the gut–bone axis and may even disrupt gut microbiota (e.g., antibiotics in PMMA), making them ill-suited to the osteoporotic microenvironment and unable to target gut dysbiosis.

Despite these advances, key challenges remain for clinical translation: (1) long-term biocompatibility and the effects of biomaterials on gut microbiota require validation in large-scale osteoporotic animal models; (2) the molecular mechanisms underlying material-gut–bone axis crosstalk require further clarification; (3) the cost-effectiveness of nanocarriers for probiotic/postbiotic delivery needs optimization; (4) personalized design based on patient-specific gut microbial composition and osteoporosis severity is critical. Future research should focus on smart, responsive systems that adapt to the dynamic osteoporotic microenvironment and gut microbiota, combining biomaterial intervention with probiotic supplementation or dietary modification to achieve synergistic therapeutic effects.

### 4.6 Critical comparison of different administration routes for biomaterials targeting gut microbiota in osteoporosis therapy

As elaborated earlier, the therapeutic efficacy of biomaterials targeting the gut microbiota for osteoporotic bone repair is closely dependent on the selection of administration routes, which directly determines targeting precision, bioavailability, safety profiles, and translational potential. A critical comparison of the three core administration routes—oral delivery, local bone delivery, and systemic delivery—is therefore indispensable to clarify their suitability for diverse clinical scenarios, identify unmet therapeutic needs, and provide guidance for the design of next-generation therapeutic strategies. We focuses on the core objective of integrating gut microbiota regulation with osteoporotic bone repair, with an emphasis on the inherent trade-offs between key performance indicators and clinical feasibility.

#### 4.6.1 Oral delivery: gut-targeted but challenged by bioavailability and specificity

Oral delivery represents the most intuitive approach for gut microbiota modulation, as it directly targets the intestinal lumen where microbial communities predominantly reside. Biomaterials designed for oral administration (e.g., nanoarmor-assisted probiotics, pH-responsive butyrate-releasing nanoparticles) are engineered to withstand the harsh gastric microenvironment (low pH and digestive enzymes) and release their cargo (probiotics, prebiotics, or metabolites) within the colon—the primary site of gut microbiota colonization (205, 222). This route offers several notable advantages: it is non-invasive, exhibits high patient compliance, and enables direct modulation of the gut microbial ecosystem, which serves as the root cause of systemic inflammation and bone metabolic disorders in osteoporosis.

However, oral delivery is associated with critical limitations that compromise its therapeutic efficacy in osteoporosis. First, bioavailability is severely constrained: even with protective encapsulation, a substantial proportion of the biomaterial cargo undergoes degradation in the upper gastrointestinal tract, thereby reducing the concentration of active agents reaching the colon. For probiotic-based biomaterials, this phenomenon translates to poor survival rates (often less than 10% of viable cells) and limited colonization of beneficial microbial taxa (e.g., Lactobacillus, Bifidobacterium) (205). Second, targeting precision is limited: oral biomaterials lack the ability to distinguish between healthy and dysbiotic gut regions, potentially inducing non-targeted modulation of microbial taxa and disrupting the native gut ecosystem. Third, oral delivery fails to directly address local osteoporotic bone defects; its effects on bone repair are indirect, relying on systemic signaling via the gut–bone axis, which may be insufficient for severe osteoporotic fractures or defects requiring localized structural support.

Clinically, oral delivery is most appropriate for mild-to-moderate osteoporosis, where systemic gut microbiota modulation constitutes the primary therapeutic goal. However, it is inadequate for patients with concurrent bone defects or those requiring rapid bone regeneration. Its non-invasive nature renders it well-suited for long-term maintenance therapy but not for acute therapeutic intervention.

#### 4.6.2 Local bone delivery: osteospecific but limited in systemic gut modulation

Local bone delivery (e.g., injectable hydrogels, modified bone cements, implantable scaffolds) is specifically designed to target osteoporotic bone defects directly, providing structural support while releasing bioactive agents (ions, prebiotics, postbiotics) that regulate local osteogenesis, angiogenesis, and immune responses (185, 190). This route offers unique advantages for osteoporosis therapy: it delivers high concentrations of biomaterials to the site of bone damage, minimizes systemic off-target effects, and synchronizes material degradation with bone regeneration—a critical feature for osteoporotic bone, which exhibits impaired remodeling capacity.

A key strength of local delivery, as highlighted earlier in the context of injectable hydrogels and nanoscale carrier systems, is its ability to indirectly link local bone repair to gut microbiota regulation: bioactive agents released at the bone site (e.g., strontium, magnesium, prebiotics) enter the systemic circulation, modulate intestinal microbial composition, and reduce systemic inflammation via the gut–bone axis (189, 220). This dual mechanism of action (local repair combined with systemic gut modulation) addresses both the local defect and the systemic root cause of osteoporosis, an advantage not afforded by conventional local bone grafts.

Nevertheless, local bone delivery is associated with critical limitations that restrict its translational potential. First, systemic gut modulation is weak and inconsistent: the concentration of bioactive agents reaching the gut is significantly lower than that at the bone site, limiting their capacity to reverse severe gut microbiota dysbiosis. For instance, prebiotics loaded in bone cements must traverse the systemic circulation to reach the colon, resulting in diluted concentrations and reduced efficacy in regulating gut microbiota (36). Second, invasiveness and donor site morbidity (for implantable scaffolds) may limit patient compliance, particularly in elderly osteoporotic patients with comorbidities. Third, local delivery is unable to address systemic osteoporotic bone loss; its effects are confined to the treated defect, necessitating combination with other administration routes for comprehensive therapy.

Local bone delivery is most suitable for patients with osteoporotic bone defects (e.g., fractures, osteonecrosis) where localized structural support and bone regeneration are prioritized. However, it requires combination with oral or systemic delivery to achieve robust gut microbiota modulation and prevent the progression of systemic bone loss.

#### 4.6.3 Systemic delivery: translational but constrained by safety and specificity

Systemic delivery (e.g., intravenous nanocarriers, colon-targeted systemic nanoparticles) involves the administration of biomaterials via the bloodstream, enabling simultaneous targeting of both the gut and bone. This route is designed to overcome the limitations of oral and local delivery: it bypasses the gastrointestinal tract (thereby improving bioavailability) and delivers bioactive agents to both systemic compartments (gut and bone) at therapeutic concentrations (189, 199, 205). For example, colon-targeted postbiotic nanoparticles administered systemically can regulate gut microbiota composition while promoting osteoblast differentiation at the bone site (195), achieving synergistic gut–bone regulation as discussed earlier in the context of nanotechnology-based delivery systems.

Systemic delivery offers several theoretical advantages: it enables precise control of drug release kinetics, targets both local bone defects and systemic gut dysbiosis, and avoids the invasiveness associated with local implants. It is particularly promising for severe osteoporosis characterized by widespread bone loss and concurrent gut microbiota dysbiosis, where multi-compartment targeting is required.

However, systemic delivery faces the most critical challenges in terms of safety and specificity. First, off-target effects are inevitable: biomaterials administered systemically may accumulate in non-target organs (e.g., liver, kidneys), increasing the risk of toxicity and reducing therapeutic efficacy. For metallic nanocarriers (e.g., gold nanospheres), long-term accumulation in the liver and spleen raises concerns regarding chronic toxicity, which has not been fully characterized in osteoporotic animal models (223). Second, cost-effectiveness is low: systemic nanocarriers require complex engineering to achieve dual gut and bone targeting, increasing production costs and limiting scalability for clinical application. Third, immune clearance of nanoscale biomaterials by the reticuloendothelial system reduces their circulation time and bioavailability, further undermining therapeutic efficacy.

Systemic delivery is currently in the preclinical stage and is not yet suitable for routine clinical use. Its potential lies in personalized therapy for severe, refractory osteoporosis, but further research is required to improve targeting precision, reduce toxicity, and optimize cost-effectiveness.

#### 4.6.4 Critical synthesis and future directions

No single administration route is optimal for all clinical scenarios; the selection depends on the severity of osteoporosis, the presence of bone defects, patient compliance, and translational feasibility (**Table 2**). Oral delivery is ideal for long-term gut microbiota modulation in mild osteoporosis but lacks the capacity for local bone repair. Local bone delivery is essential for defect repair but requires combination with other routes for effective systemic gut regulation, as noted earlier in the discussion of injectable hydrogels and modified bone cements. Systemic delivery offers multi-compartment targeting but is limited by safety concerns and cost constraints.

**Table 2:** Critical comparison of administration routes for gut microbiota-targeted biomaterials in osteoporosis therapy.

Administration route	Key advantages	Critical limitations	Optimal clinical scenario
Oral Delivery	**Non-invasive; high patient compliance; direct gut targeting; suitable for long-term therapy**	**Low bioavailability; limited targeting precision; lack of local bone repair capacity**	**Mild-to-moderate osteoporosis; long-term gut microbiota maintenance**
Local Bone Delivery	**High local bioavailability; direct bone defect repair; synchronized regeneration; low systemic toxicity**	**Weak and inconsistent systemic gut modulation; invasiveness (for implants); inability to address systemic bone loss**	**Osteoporotic bone defects (fractures, osteonecrosis); localized repair requirements**
Systemic Delivery	**Multi-compartment targeting (gut + bone); precise control of drug release; bypasses gastrointestinal degradation**	**Inevitable off-target toxicity; high cost; immune clearance; limited to preclinical stage**	**Severe, refractory osteoporosis; concurrent gut dysbiosis and systemic bone loss**

A critical unmet therapeutic need, aligned with the future perspectives outlined earlier, is the development of hybrid delivery systems that integrate the strengths of multiple routes—for example, local bone scaffolds capable of releasing gut-targeted agents into the systemic circulation, or oral nanocarriers modified to target bone via surface functionalization. Such systems would address both local defects and systemic gut dysbiosis, overcoming the limitations of single administration routes. Additionally, personalized delivery strategies—tailored to patient-specific gut microbial composition and osteoporosis severity—are required to optimize therapeutic efficacy and reduce off-target effects.

In summary, each administration route exhibits distinct trade-offs between targeting precision, bioavailability, safety, and clinical feasibility. A critical understanding of these trade-offs is essential to guide the design of biomaterials that effectively integrate gut microbiota regulation with osteoporotic bone repair, thereby accelerating translational progress in this emerging field.

## 5 Conclusion and perspectives

In conclusion, emerging biomaterials targeting the gut microbiota have emerged as a transformative therapeutic strategy for osteoporotic bone repair, bridging the gap between local bone defect regeneration and systemic gut microbiota regulation that conventional bone repair materials fail to address. This chapter systematically summarizes the design principles, therapeutic mechanisms, and critical applications of modified bone cements, injectable hydrogels, nanoscale carrier systems, and immuno-active biomaterials, all of which integrate local osteogenic, angiogenic, and immunomodulatory functions with systemic gut–bone axis regulation to achieve synergistic therapeutic effects in osteoporosis. A critical comparison of oral, local bone, and systemic administration routes further clarifies the inherent trade-offs between targeting precision, bioavailability, safety, and clinical feasibility, providing essential guidance for the rational selection of delivery strategies based on clinical needs. Collectively, these biomaterial-based approaches leverage the “material–microbiota–metabolism–bone” axis to reverse gut microbiota dysbiosis, alleviate systemic chronic inflammation, and promote osteoporotic bone regeneration, offering a novel paradigm that transcends the limitations of traditional bone-centered interventions. Looking forward, future research should focus on addressing the current challenges, including improving the targeting precision of biomaterials, optimizing the long-term biocompatibility and ecological safety of degradation by-products, and developing personalized hybrid delivery systems that integrate the strengths of multiple administration routes. The integration of multi-omics technologies, artificial intelligence, and biomimetic *in vitro* models (e.g., gut-on-chip platforms) will further deepen the understanding of material–gut microbiota–bone crosstalk mechanisms, accelerating the translational progress of these innovative strategies from preclinical research to clinical application, and ultimately providing more effective therapeutic options for the prevention and treatment of osteoporosis.
